# Critical assessment of the comparison of needle gauges for fine needle aspiration of lymph nodes and thyroid

**DOI:** 10.1097/MS9.0000000000004754

**Published:** 2026-01-21

**Authors:** Baneen Javaid Arain, Sidrah Anwar, Hermann Yokolo

**Affiliations:** a Department of Medicine, Jinnah Sindh Medical University, Karachi, Pakistan; b Department of Research, Medical Research Circle (MedReC), Goma, DR Congo

*Dear Editor,*

We read with interest the article by Alzahrani, M. Y., *et al*, entitled “Comparison of fine-needle aspiration cytology with 22- and 25-gauge needles in thyroid and lymph node lesions”^[1]^. This study provides relevant data on the impact of needle gauge on cytological adequacy and smear quality. However, certain methodological and analytical limitations warrant discussion to refine the interpretation. This letter aims to highlight these points and formulate recommendations that could improve the statistical robustness and clinical applicability of future research. This article is consistent with the TITAN 2025 guidelines governing the use of AI^[2]^.

A key limitation lies in the statistical analysis, where multiple outcomes (adequacy, bloodiness, conclusiveness) were analyzed independently using a conventional significance threshold of *P* < 0.05, without adjustment for multiple comparisons. This omission substantially increases the risk of type I error and reduces the reliability of marginally significant results. More rigorous approaches, such as the application of adjusted significance thresholds demonstrated in other comparative studies, would have strengthened the validity of their findings. Additionally, cytological assessment was performed without blinding to the needle gauge, introducing potential observer bias. Since knowledge of the instrument used can consciously or unconsciously influence the interpretation of smear quality, the absence of blinding further undermines the objectivity of the results^[3]^.

Second, in this investigation, cytological adequacy was solely assessed in binary terms using the Bethesda framework, classifying smears as adequate or inadequate. This simplified approach does not account for variations in specimen quality, such as cellularity, colloid content, or blood contamination; therefore, a sample could meet adequacy criteria yet still be diagnostically suboptimal. As a result, subtle but clinically important variations between 22G and 25G needles may have been overlooked, explaining the lack of significant findings. In contrast, studies utilizing more detailed morphologic scoring systems that grade numerous cytologic aspects have demonstrated higher sensitivity in detecting such variations, emphasizing the need for more granular evaluation methods in future studies^[4]^.

Thirdly, the study’s omission of patient-reported outcomes, like pain and procedural distress, limits the assessment of clinical efficacy. For example, Cianci *et al* (2024) compared 22G and 25G needles in ultrasound-guided thyroid Fine Needle Aspiration (FNA), reporting that 98.7% of patients in the 25G group experienced only mild discomfort [Visual Analog Scale (VAS) 0–5], compared to 90% in the 22G group, which also reported cases of severe pain (VAS 6–10)^[5]^. Incorporation of a VAS would offer a more patient-centered evaluation, potentially providing support for evidence-based needle selection, thereby optimizing the balance between diagnostic adequacy and procedural comfort.

Fourth, the study’s primary focus is on cytological adequacy, while underscoring the need for histopathological correlation. Cytological adequacy may not equate to diagnostic accuracy. A conclusive cytological result, if not verified using surgical histopathology, may still represent a false negative. To ensure diagnostic validity and clinical applicability, future investigations should validate cytological findings against corresponding histopathological outcomes, as demonstrated by Gąsiorowski *et al*^[6]^.

Lastly, the current presentation of adequacy data lacks per-pass or cumulative metrics, limiting the depth across successive passes provided. Kuzan *et al* findings illustrated that adequacy improved progressively with each additional pass (76.0, 87.6, 90.1, and 91.7%), with a subsequent plateau beyond the third pass – supporting a data-driven recommendation of two to three passes^[7]^. The integration of per-pass adequacy tables coupled with statistical comparisons such as chi-squared tests or logistic regression, in future studies, would enable readers to delineate the point of diminishing returns and refine procedural efficiency.

In summary, while the authors’ work contributes valuable data on needle gauge and specimen quality, incorporating standardized pain assessment, histopathological validation, and cumulative adequacy analysis would substantially enhance the robustness and clinical relevance of future research on thyroid and lymph-node FNAs.

## Data Availability

Not applicable.
